# A Low-Cost Ultrasound Program Leads to Increased Antenatal Clinic Visits and Attended Deliveries at a Health Care Clinic in Rural Uganda

**DOI:** 10.1371/journal.pone.0078450

**Published:** 2013-10-30

**Authors:** Andrew B. Ross, Kristen K. DeStigter, Matthew Rielly, Sonia Souza, Gabriel Eli Morey, Melissa Nelson, Eric Z. Silfen, Brian Garra, Alphonsus Matovu, Michael Grace Kawooya

**Affiliations:** 1 Department of Radiology, University of Vermont College of Medicine, Fletcher Allen Health Care, Burlington, Vermont, United States of America; 2 Ultrasound Research and Development, Philips Healthcare, Philips Innovation Campus, Bangalore, Karnataka, India; 3 Research Division, Philips Healthcare, Bothell, Washington, United States of America; 4 University of Vermont College of Medicine, Fletcher Allen Health Care, Burlington, Vermont, United States of America; 5 Philips Health Care, Andover, Massachusetts, United States of America; 6 Department of Radiology, Veterans Affairs Hospital, Washington, DC, United States of America; 7 Department of Surgery, Kamuli Mission Hospital, Kamuli, Uganda; 8 Ernest Cook Ultrasound Research and Education Institute, Mengo Hospital, Kampala, Uganda; Iran University of Medical Sciences, Iran (Islamic Republic Of)

## Abstract

**Background:**

In June of 2010, an antenatal ultrasound program to perform basic screening for high-risk pregnancies was introduced at a community health care center in rural Uganda. Whether the addition of ultrasound scanning to antenatal visits at the health center would encourage or discourage potential patients was unknown. Our study sought to evaluate trends in the numbers of antenatal visits and deliveries at the clinic, pre- and post-introduction of antenatal ultrasound to determine what effect the presence of ultrasound at the clinic had on these metrics.

**Methods and Findings:**

Records at Nawanyago clinic were reviewed to obtain the number of antenatal visits and deliveries for the 42 months preceding the introduction of ultrasound and the 23 months following. The monthly mean deliveries and antenatal visits by category (first visit through fourth return visit) were compared pre- and post- ultrasound using a Kruskal-Wallis one-way ANOVA. Following the introduction of ultrasound, significant increases were seen in the number of mean monthly deliveries and antenatal visits. The mean number of monthly deliveries at the clinic increased by 17.0 (13.3–20.6, 95% CI) from a pre-ultrasound average of 28.4 to a post-ultrasound monthly average of 45.4. The number of deliveries at a comparison clinic remained flat over this same time period. The monthly mean number of antenatal visits increased by 97.4 (83.3–111.5, 95% CI) from a baseline monthly average of 133.5 to a post-ultrasound monthly mean of 231.0, with increases seen in all categories of antenatal visits.

**Conclusions:**

The availability of a low-cost antenatal ultrasound program may assist progress towards Millennium Development Goal 5 by encouraging women in a rural environment to come to a health care facility for skilled antenatal care and delivery assistance instead of utilizing more traditional methods.

## Introduction

### Background

In 2000, 189 member states came together to sign the United Nations Millennium Declaration, a road map of ambitious goals to address the many faces of poverty widely prevalent in the world's developing nations. Improving maternal health became Millennium Development Goal 5 (MDG 5) with a focus on decreasing maternal mortality and increasing access to family planning [1]. Two targets were selected to monitor progress towards MDG 5 by the 2015 deadline: 1) reducing the maternal mortality ratio (MMR, defined as number of maternal deaths per 100000 live births) by 75% and 2) increasing the proportion of births attended by skilled health care personnel by 90%.

Despite this effort, concern has been expressed over the feasibility of achieving MDG 5 by the 2015 target [2]–[4]. The challenges identified are numerous and varied but include inadequate health care systems, low numbers of skilled birth attendants, high fertility rates, and low levels of education. These problems are most acute in the rural areas of the developing world leading to staggering inequities in the burden of maternal death. For example, in 2010 the number of maternal deaths per 100000 live births in Sub-Saharan Africa was estimated at 500 (400–750, 95% CI), an astonishing 30 times greater than the estimate of MMR for developed countries in the same year [5]. The need for continued investigation into effective interventions is acute.

### Skilled Health Care at Delivery, a Key Strategy to Reduce MMR

A strategy that has shown consistent promise in reducing MMR is attendance at birth by skilled health care personnel [6], [7]. This number has been easier to track than MMR and is used as a proxy indicator in marking progress toward MDG 5. Skilled care during the antenatal, peri-partum, and post-partum period is an explicit priority of the global health community with all countries asked to ensure access to such care in a joint statement from the WHO, UNFPA, UNICEF, and World Bank [8]. The majority of maternal deaths occur in the intrapartum period and many of these cannot be anticipated [3]. A skilled birth attendant (SBA) working within a health care system which provides an avenue for referral and management of obstetric emergencies can often successfully manage the most common causes of peripartum maternal death, listed in descending order by the World Health Organization as hemorrhage, sepsis, unsafe abortion, eclampsia, and obstructed labor [9]. Current evidence suggests that as countries increase the availability of skilled birth attendance, maternal mortality will decline and this has been shown to be a more successful strategy than relying on the training of traditional birth attendants [10], [11]. Policies to increase the number of deliveries attended by SBAs remain an important focus of international efforts to decrease MMR [12].

The effectiveness of antenatal care in reducing maternal mortality has been and continues to be controversial [13] but remains an important component of efforts to decrease both maternal mortality and perinatal death, particularly in the developing world. Antenatal care (ANC) visits allow opportunities for maternal education regarding safe birthing and home care practices including umbilical stump hygiene, lactation practices, and under what circumstances to seek skilled medical care outside the home or village environment. Developing relationships with pregnant women during an antenatal visit may also increase the likelihood of having a skilled birth attendant present at delivery. Practical strategies to encourage ANC visits and skilled care at delivery remain an important area of investigation to inform international health policy efforts.

### Uganda Health Background

Uganda is a country of approximately 34 million people with a high annual birth rate, exceeding the average for Sub-Saharan Africa with an annual population growth of 3.2% [14]. Like most of Sub-Saharan Africa, Uganda has a high burden of maternal mortality. Although overall there has been increasing availability to health care over the last decade-in 2010, 72% of the population lived within 5 km of a health care facility compared with 49% in 2000-access to skilled maternal care remains split with the poor and those living in rural areas significantly less likely to have access to health care resources [15]. Maternal mortality ratio has decreased over the last 20 years from 600 per 100,000 live births in 1990 to 310 in 2010 [16]. Nonetheless this remains unacceptably high with women facing a 1 in 49 lifetime risk of dying during childbirth. In the United States the lifetime risk is 1 in 2,400 [5].

Public health efforts in Uganda have focused on increasing attendance at delivery by a skilled health care provider, frequently a nurse midwife. Many of these providers are trained in the Active Management of the Third Stage of Labor program, a set of recommendations to prevent postpartum hemorrhage that remains the leading cause of maternal mortality resulting in 35% of deaths [16]. Particularly in the rural areas of Uganda simply encouraging women to give birth with a skilled birth attendant remains a challenge, with the overall percentage of attended births having only increased slightly from 38% in 1989 to 42% in 2006 and women in the top quintile of household income more than twice as likely to have a skilled provider present at delivery than women in the bottom quintile [16].

### The Role of Ultrasound in Pregnancy

Most previous investigations into the use of routine antenatal ultrasound have taken place in the developed world where full size ultrasound machines are used by trained sonographers. Radiologists and obstetricians interpret the images and access to the necessary follow-up is typically available. This type of high cost program has been found to be unfeasible in the low resource setting. Work by Geerts et al. evaluated a mix of high risk urban and rural patients at a hospital in South Africa and demonstrated only a small benefit; specifically fewer women were induced for post-dates delayed labor with the more accurate pregnancy dating afforded by ultrasound [17]. This small benefit was not felt to be worth the large cost of the program. Van Dyk et al. found similar results in a more recent study also based in South Africa [18]. The study was hampered by a high percentage of study participants lost to follow up but found no benefit on rates of miscarriage, perinatal death, prenatal hospitalization, or low birth weight. The number of study participants did not allow adequate statistical power to examine maternal mortality.

Although previous research on antenatal ultrasound has shown only a small benefit with questionable cost-effectiveness, these studies have mostly taken place in developed countries and, even when done in a low resource setting, have looked at high-cost, comprehensive ultrasound screening programs. There is a need for further research into the use of low-cost ultrasound programs focused on identifying high-risk conditions of pregnancy as an early warning system to allow for appropriate triage and referral of patients at greatest risk for poor outcomes. However, the broader picture of introducing ultrasound into a rural, low resource environment must also be considered. In this context, where few skilled health care providers are available, the alternative to seeking antenatal care and delivery assistance at a clinic is typically delivery at home under the care of a traditional birth attendant. Thus, if global public health goals of increasing the presence of an SBA at delivery are to be met, the technology must be acceptable to patients and not discourage them from seeking care at a clinic.

There has been prior research regarding the acceptability of ultrasound to mothers in low resource settings. Rijken et al. found that women on the Thai-Burmese border were likely to view ultrasound favorably as a technology that could increase safety during pregnancy and delivery [19]. However there has also been concern for overuse of ultrasound scanning during pregnancy with multiple follow up scans recommended, potentially as a way for private clinics to increase revenue [20], [21]. Additionally, there has been concern about disclosing the fetal sex [22]. Clearly the introduction of antenatal ultrasound scanning into a low resource and ultrasound naive setting must be done cautiously.

A consensus framework for evaluating the use of technology in developing countries has been proposed with the following parameters: impact on improving health, appropriateness and cultural acceptability, feasibility, decreasing the knowledge gap, and the provision of other indirect benefits to the community [23]. To succeed a technology must not only be effective but also acceptable to the local community's traditions and attitudes. Whether the addition of ultrasound scanning to ANC visits would encourage or discourage potential patient visits was an unknown question of critical interest. Our study specifically investigates the effect of a low cost antenatal ultrasound program on the number of women coming to a health care clinic in rural Uganda for ANC visits and for skilled care at delivery.

## Methods

In June 2010 a program was put in place by the NGO Imaging the World (ITW; www.imagingtheworld.com) to provide access to basic antenatal ultrasound at the Nawanyago community level III health care centre (HC III) in Uganda. Women are offered an ultrasound scan at the first ANC visit, and again at 32 weeks gestation. If there is a clinical indication (infections, hemorrhage, pain), then scans are done at other ANC visits as well. With a population of 23,000, the health care needs of Nawanyago sub-county in Kamuli District are served by this private HC III (under the jurisdiction of the Diocese of Jinja) in addition to a government HC III in Bupadhengo, 4 km away. Services offered at the clinic by two midwives with many years of experience include antenatal care visits, testing and treatment for co-morbidities of pregnancy (malaria, worms, anemia, hypertension, HIV, syphilis, etc.), and skilled routine vaginal deliveries (including breech deliveries). Most pregnant women requiring urgent or emergent care, including C-section, are referred to Kamuli Mission Hospital, a distance of 24 km from Nawanyago HC III.

To address the human resource problem of few trained sonographers in low resource settings, scanning protocols have been developed by ITW that rely solely on the use of surface anatomy landmarks. The ultrasound probe is passed across the pregnant abdomen in a series of six prescribed sweeps acquiring a series of static images. A low-frequency curved transducer is used to obtain the sweeps with the pregnant patient in a supine position, having a full bladder. This volume of images can be scrolled through by the reviewer like a short video. An initial feasibility study confirmed that the images obtained with this protocol are of diagnostic quality [24]. Low cost, portable ultrasound machines are utilized. The machines have been preconfigured to provide three presets to optimize image quality for three BMI categories: small, medium, or large. The acquired images are de-identified and stored locally on a laptop computer before being compressed and transmitted digitally via cell phone modem to a remote internet server where they can then be accessed by a credentialed reviewer, either in country or abroad, for interpretation. An abbreviated report of the findings is sent via SMS to the nurse midwife's cell phone with the full report sent by email. Women are thus able to receive the initial results of their scan prior to the end of the visit, and they are given recommendations for the next steps in their care. The fetal gender is not disclosed. The full program methodology has been previously reported [25].

Two midwives from Nawanyago HC III were trained in the protocol by ITW over a 3-day period. The educational program included classroom and hands-on teaching with assessment of competency. Final interpretations were given by credentialed sonographers from Kamuli Mission Hospital, the primary referral hospital for Nawanyago HC III. During the period of the study, quality assurance for both image quality and accuracy of interpretations was performed remotely by ITW. Although Ugandan ITW personnel made weekly site visits, foreign ITW volunteers re-visited the site only a total of 6 days during the pilot study period. A small fee (approximately $2 USD) was charged per exam to help with the clinic operating costs and to remunerate the interpreters. The fee was determined by the clinic based on what was felt to be affordable to the local community. The revenue generated allows the program to be financially self-sustaining. All patients were able to afford the fee for the ultrasound examination.

Ultrasound can diagnose many of the most common causes of maternal and neonatal mortality including multiple gestations, sequelae of abortion, causes of obstructed labor, and specific causes of maternal hemorrhage such as placenta previa [9], and this is the focus of the ITW program. The implemented scanning protocols allow for reliable identification of fetal presentation and number as well as placental position. Early identification of high-risk pregnancies allows clinic staff to recommend delivery with skilled care at the clinic instead of at home or timely referral to the district hospital in Kamuli for a higher level of obstetric care. All patients who were referred to Kamuli Mission Hospital for definitive diagnosis and treatment made the journey.

Originally, the ultrasound scanning protocols were evaluated through an IRB-approved concordance study that assessed the effectiveness of the ITW methods as previously reported [25]. While evaluating the data we observed certain clinical trends in the ANC visit and delivery data that merited further consideration. Using the existing dataset, we developed a retrospective study protocol to evaluate the “magnet effect” associated with the introduction of antenatal ultrasound scanning at the HC III.

Following introduction of the ultrasound program in June of 2010, records at the HC III, including number of ANC visits and deliveries, were collected through April of 2012. Historical control data obtained from January 2007 through the introduction date were used as comparator. Data included the 41 months preceding the introduction of the ultrasound program and the 23 months following. For the historical control, only aggregated, clinic-level data was available. This constituted data available from clinic records that are updated by clinic staff and kept on hard copy at the clinic in a secure location. This data is also reported to the Diocese of Jinja and the Uganda Ministry of Health and is publicly available in aggregated form. The available information included: the number of births at the clinic per month, the number of first ANC visits per month, the number of second ANC visits per month, the number of third ANC visits per month and the number of fourth ANC visits per month. All clinic records were subsequently audited by an independent research associate from the Makerere University School of Public Health in Kampala and found to be consistent with the numbers provided to the research team by clinic staff.

Statistical analysis was performed using SAS version 9.3 (SAS Inc., Cary, NC, USA). For the historical control data, for both deliveries and ANCs were available as aggregated monthly counts. For comparability, the data collected after the introduction of ultrasound were collapsed into aggregated monthly counts as well. The number of ANCs was examined both categorized by number of visit (first time ANC visit through fourth return ANC visit) and in sum as total number of ANCs. Results were summarized descriptively using the number of observations, mean, standard deviation (SD), 95% confidence interval (CI) for the mean, median, minimum, and maximum values.

Prior to statistical testing, the data were assessed for normality by visual inspection of plots and formally by the Shapiro-Wilk's test. In all cases, the data were not normally distributed; therefore non-parametric statistical analyses were used. A Kruskal–Wallis one-way analysis of variance (KW-ANOVA) by ranks was conducted to compare the data from the pre-ultrasound time period to the post-ultrasound time period.

Due to the retrospective nature of the study, the validity of the historical control was assessed. A KW-ANOVA was conducted to determine if the data were consistent over time prior to the introduction of ultrasound, and if the data were consistent over time after the introduction of ultrasound. Each model included one factor for year. If the model indicated that the data were consistent over time prior to the introduction of ultrasound (i.e., year was not a significant factor) and the data were consistent after the introduction of ultrasound, it could be interpreted that no confounding event occurred prior to or after ultrasound introduction (June 2010) to alter the number of deliveries or ANC visits.

Additionally, the number of deliveries during the months from July 2008 to June 2012 from a nearby (4 km) government facility (Bupadhengo HCIII) was available. These data were compared pre-June 2010 to post June-2010. If the number of deliveries remained stable over time, it would further support the assumption that no confounding event occurred in the general Nawanyago area in June 2010 that altered the number of deliveries.

### Ethics Statement

Investigational Review Board (IRB) approval was obtained from the Mengo Hospital Research Review Committee, (IRB title Evaluation of Simple Ultrasound Protocols for Improving Access to Ultrasound in Low Resource Settings, study number 013/05–10). Written informed consent was obtained from all study participants with appropriate translation and literacy resources provided as necessary. The consent form and process were approved by the IRB. All ultrasound images and patient records were de-identified with the patients receiving an “ITW number” that functioned as the medical record number. Patient identifying information was kept in a secure location at Nawanyago HC III. All Data were coded anonymously for analysis. Ultrasound results were recorded in the Ugandan Ministry of Health Maternal Passport, which is distributed by the HC III to all pregnant patients. For ethical reasons, fetal gender determination is not performed and the program is periodically audited to ensure compliance with this requirement.

## Results

**Figure 1:**
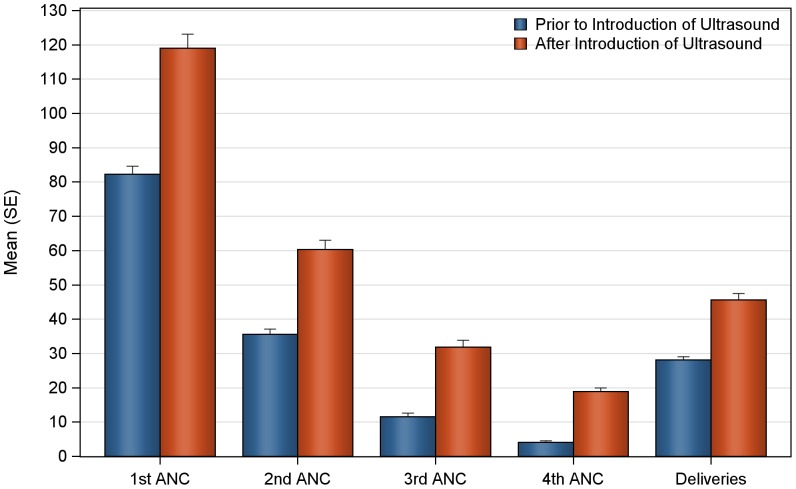
Number of deliveries and antenatal care visits prior to and after introduction of ultrasound at Nawanyago Health Center III.

The mean number of deliveries and ANC visits prior to, and after introduction of ultrasound are summarized in Figure 1 and Table 1. Statistically significant increases were seen in the monthly mean number of deliveries and ANC visits following the introduction of the antenatal ultrasound program at Nawanyago (KW-ANOVA p-value<0.0001 in all cases). The mean number of monthly deliveries at the clinic prior to ultrasound was 28.1 (SD 6.01) that significantly increased to a post-ultrasound program mean of 45.6 (SD 8.92). Total ANC visits also demonstrated a significant increase following the introduction of ultrasound with a rise from 133.5 (SD 25.91) to 230.3 (SD 27.62). Increases were observed across all categories of ANC visits, with the number of first ANC visits increasing from 82.2 (SD 15.66) to 119.1 (SD 19.51); second ANC visits increased from 35.6 (SD 9.93) to 60.4 (SD 12.46); third ANC visits increased from 11.6 (SD 6.48) to 31.9 (SD 9.44) and fourth return ANC visits increased from 4.1 (SD 2.71) to 19.0 (SD 5.06).

**Table 1:** Number of monthly deliveries and antenatal care visits at Nawanyago Health Center III pre and post ultrasound program.

Endpoint	Time	N	Mean (SD)	Median	Min–Max	95% CI
Number of Attended Deliveries	Prior to Introduction of Ultrasound	41	28.1 (6.01)	29.00	15.0–40.0	(26.2, 30.0)
	After Introduction of Ultrasound	23	45.6 (8.92)	47.00	26.0–58.0	(41.8, 49.5)
Total Number of Antenatal Visits	Prior to Introduction of Ultrasound	41	133.5 (25.91)	132.00	69.0–180.0	(125.4, 141.7)
	After Introduction of Ultrasound	23	230.3 (27.62)	225.00	181.0–271.0	(218.4, 242.2)
First Antenatal visit	Prior to Introduction of Ultrasound	41	82.2 (15.66)	80.00	57.0–126.0	(77.3, 87.2)
	After Introduction of Ultrasound	23	119.1 (19.51)	121.00	88.0–163.0	(110.7, 127.5)
Second Antenatal visit	Prior to Introduction of Ultrasound	41	35.6 (9.93)	35.00	4.0–55.0	(32.4, 38.7)
	After Introduction of Ultrasound	23	60.4 (12.46)	62.00	27.0–76.0	(55.0, 65.8)
Third Antenatal visit	Prior to Introduction of Ultrasound	41	11.6 (6.48)	10.00	0.0–31.0	(9.6, 13.7)
	After Introduction of Ultrasound	23	31.9 (9.44)	30.00	17.0–49.0	(27.8, 36.0)
Fourth Antenatal visit	Prior to Introduction of Ultrasound	41	4.1 (2.71)	4.00	0.0–11.0	(3.3, 5.0)
	After Introduction of Ultrasound	23	19.0 (5.06)	19.00	10.0–32.0	(16.8, 21.1)

**Figure 2:**
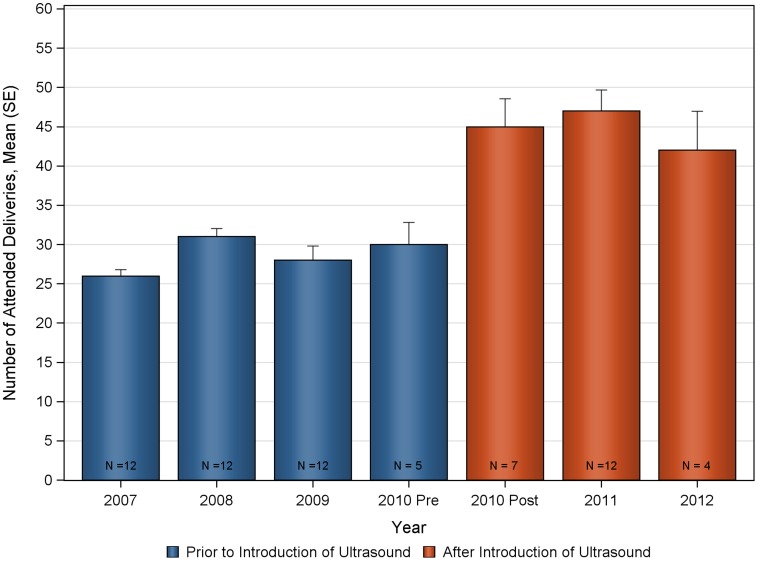
Number of deliveries over time at Nawanyago Health Center III. N represents the number of months included in the calculation.

**Figure 3:**
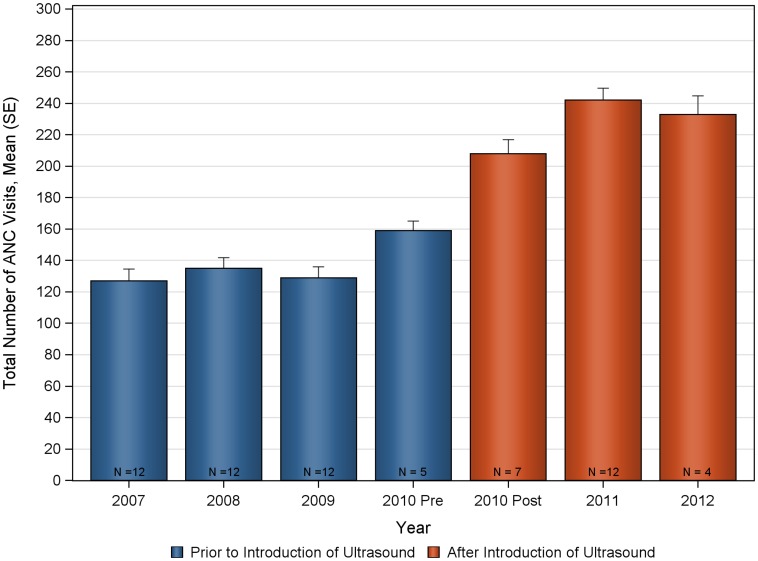
Total number of antenatal care visits over time at Nawanyago Health Center III. N represents the number of months included in the calculation.

**Figure 4:**
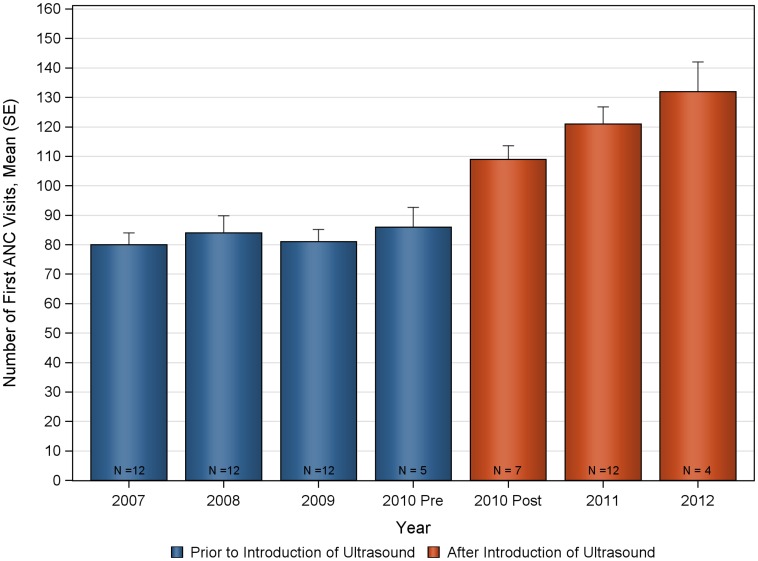
Number of first antenatal care visits over time at Nawanyago Health Center III. N represents the number of months included in the calculation.

**Figure 5:**
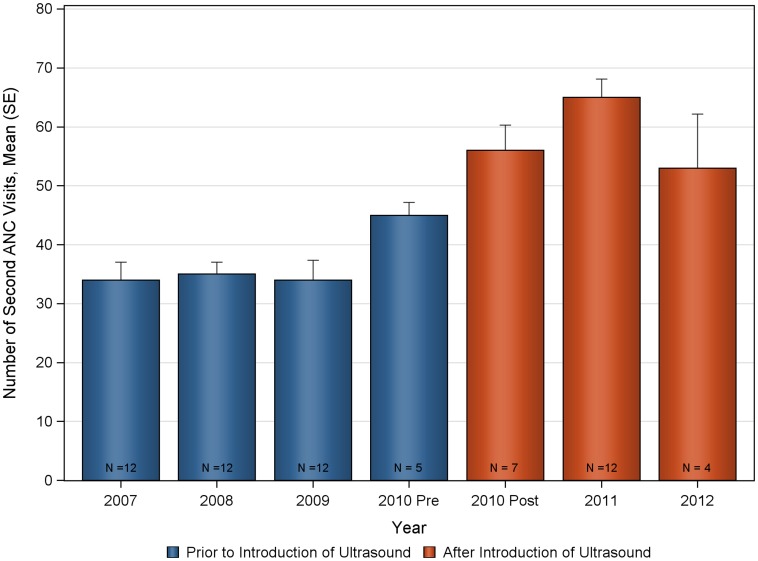
Number of second antenatal care visits over time at Nawanyago Health Center III. N represents the number of months included in the calculation.

**Figure 6:**
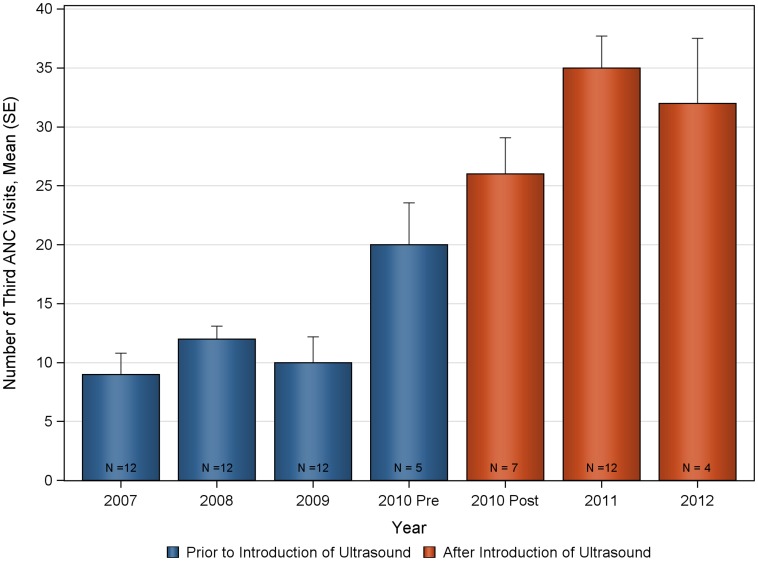
Number of third antenatal care visits over time at Nawanyago Health Center III. N represents the number of months included in the calculation.

**Figure 7:**
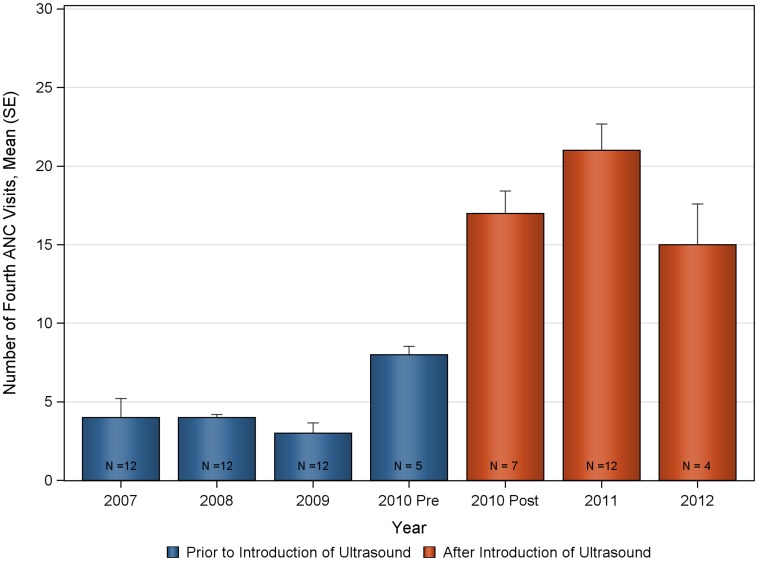
Number of fourth antenatal care visits over time at Nawanyago Health Center III. N represents the number of months included in the calculation.

The deliveries and ANC visits data were assessed for consistency both for the pre and post ultrasound time period to evaluate the appropriateness of the historical control (Figures 2, 3, 4, 5, 6, 7). Taken separately, there was no significant difference in the number of attended deliveries over time prior to the introduction of ultrasound (KW-ANOVA p-value = 0.1482), or after the introduction of ultrasound (KW-ANOVA p-value = 0.6213). Similarly, there was no significant difference in the number of first or second ANC visits over time prior to the introduction of ultrasound (KW-ANOVA p-value = 0.8263 and 0.0656, respectively). However there was a significant difference over time in the number of third and fourth ANC visits prior to the introduction of ultrasound (KW-ANOVA p-value = 0.0432 and 0.0213, respectively). The first 5 months of year 2010 seem to have higher number of ANC3 and ANC4 visits compared to 2007, 2008 and 2009; however the sample size is smaller for 2010 and this may contribute to the observed difference. Also, the ultrasound machine was not physically installed until June, however the announcement concerning the ITW presence was done early in 2010, and a major stakeholder meeting was held in April 2010. The publicity could have prompted women to come to the clinic in anticipation of the project.

**Figure 8:**
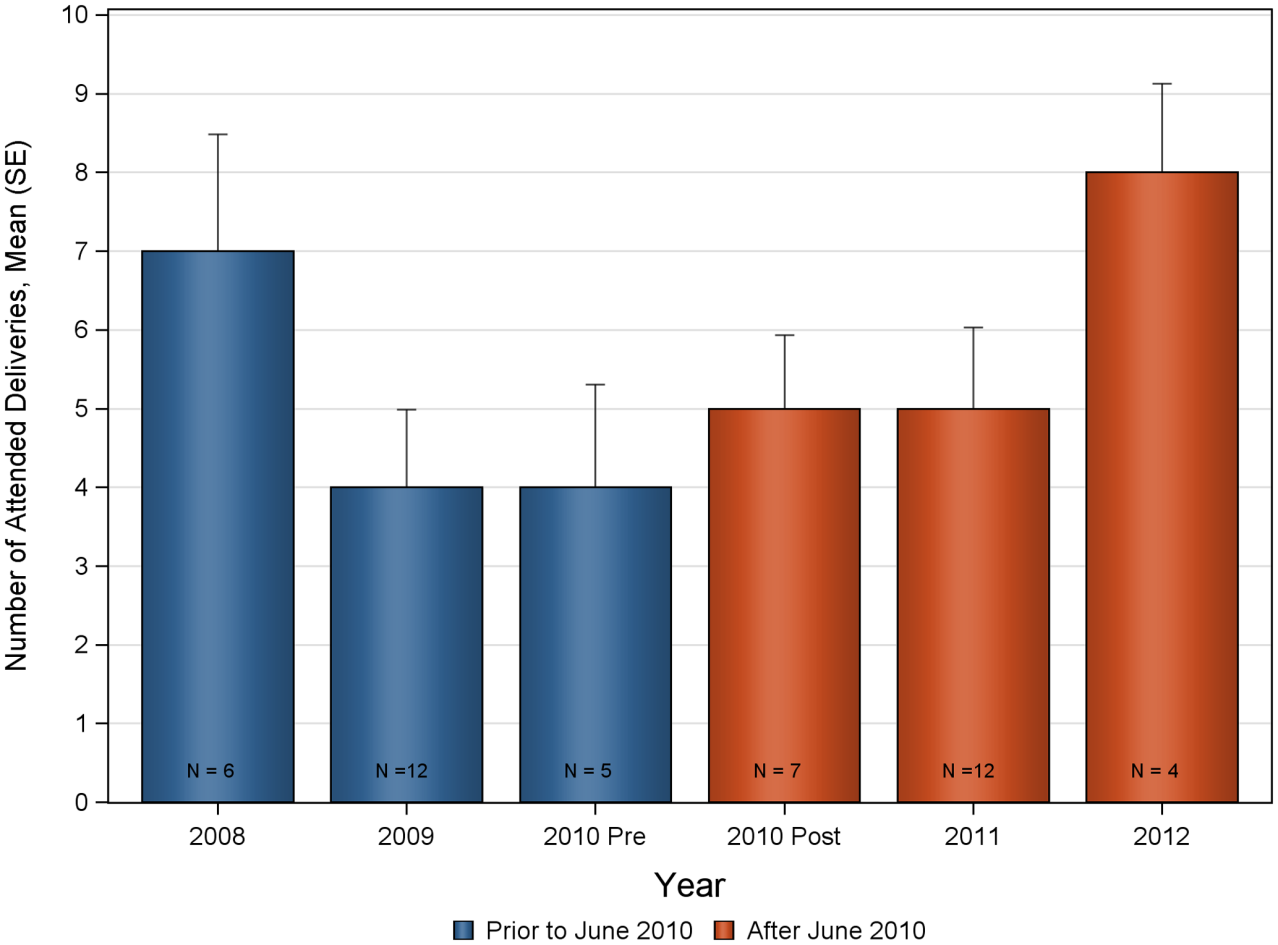
Number of deliveries over time at Bupadhengo Health Center III. N represents the number of months included in the calculation.

Overall, the results prior to the introduction of ultrasound are consistent, and the results after the introduction of ultrasound are consistent. As the information was collected at only one clinic, all other standards of care were stable. Additionally, the patient population is assumed to be unchanging over time. Summary statistics for the mean number of deliveries at Bupadhengo HCIII before and after June 2010 are presented in Table 2. The mean number over time is presented in Figure 8. Analysis of the Bupadhengo HCIII data indicated that there was no change in the number of deliveries over time for the period from July 2009 to May 2010 (KW-ANOVA p-value = 0.2301) and the mean number of deliveries was 5.0 (SD 2.9); or for the time period from June 2010 to April 2012 (KW-ANOVA p-value = 0.3008) and the mean number of deliveries was 5.5 (SD 3.1). When comparing the overall time period prior to June 2010 to post-June 2010, the number of deliveries was consistent over time (KW-ANOVA p-value = 0.4933). All of these conditions support the use of 2007–2010 data as the historical control for these analyses.

**Table 2:** Number of monthly deliveries at Bupadhengo Health Center III pre and post June 2010.

Endpoint	Time	N	Mean (SD)	Median	Min–Max	95% CI
Number of Attended Deliveries	Prior to June 2010	23	5.0 (2.95)	5.00	1.0–14.0	(3.7, 6.2)
	After June 2010	23	5.5 (3.13)	5.00	0.0–11.0	(4.1, 6.8)

## Discussion

Our results indicate a significant increase in the number of deliveries and ANC visits associated with the introduction of an antenatal ultrasound program at Nawanyago HCIII. The increase in the number of deliveries is particularly significant because although most Ugandan women (94%) have at least one ANC visit, much fewer (41%) deliver in a health facility [14]. The added knowledge about the status of the pregnancy provided by the integration of ultrasound into the ANC visits may be instrumental in guiding decisions about where to give birth. This has important implications for assisting progress towards MDG 5 by increasing the number of women giving birth with an SBA instead of at home without the assistance of a trained caregiver. Anecdotal impressions from women's comments at the clinic suggest that the presence of ultrasound technology increased trust in the health care system. An additional observation was that husbands were more likely to accompany their wives to ANC visits because they were interested in watching the ultrasound scan (the “TV”). This could be included as a measurable data point in a future study. Husbands are frequently the health care decision makers in Uganda [26] and it is possible that their increased involvement in pregnancy care may in part explain the observed “magnet effect” of the antenatal ultrasound program. Previous studies have found that women in low resource countries perceive ultrasound positively, as a chance to view the baby and receive reassurance that the pregnancy is progressing normally. They also reacted well to education on pregnancy as a part of these visits [20]. This may play an important role in attracting women to the clinic.

The increase in ANC visits is likely to also have a significant direct impact on maternal and neonatal morbidity and mortality, given the services that are routinely provided at these visits, including testing for HIV, IPT for malaria, deworming, providing iron and folate, testing blood pressure, and offering education for nutrition, mosquito nets and post-natal infant care. It is therefore possible that the “magnet effect” of ultrasound at the lower level health facility results in an overall improved quality of maternal and newborn care.

However, these results must be interpreted with caution given a number of study limitations. Research in rural Africa faces many challenges. Records may be incomplete, unavailable, or unreliable. Travel to field sites is difficult and time consuming. A poor population with limited health care resources requires balancing the needs of research with basic humanitarian considerations. This trial would have been better served with an external control site, and although a comparable site was sought, each site considered either had inadequate records or was involved with the activities of another NGO. Thus an historic control was necessary and the presence of confounding must be carefully evaluated. It should be noted that the workflow at the clinic remained the same both before and after the introduction of ultrasound with a recommendation of four ANC visits as per WHO guidelines. Thus there was no artificial inflation of ANC visits by an altered workflow. Other than ITW, there were no other NGOs operating in Nawanyago sub-county during the ultrasound program. The ultrasound program was accompanied by an increased number of visits from Imaging the World volunteers and local team members, and it should be acknowledged that this may account for part of the observed effect. However, foreign personnel were on site for a total of only six days during the study period. Additionally, the increase in ANC visits and deliveries persisted for a nearly two year follow up period. Infrastructure at the clinic remained unchanged for the period of the study. No specific improvements were made to accommodate the ultrasound machine. The local political conditions and environment were also grossly stable as best as could be determined from clinic staff. In short, although the possibility for residual confounding remains, the increase in deliveries and ANC visits was both statistically and clinically significant, occurred shortly after the introduction of the ultrasound program, and was maintained throughout the follow up period. Therefore it seems plausible that the observed effect was a direct result of ultrasound availability at the clinic.

It will be important to determine whether, in this rural resource-constrained setting, ultrasound plays a role in reducing neonatal and maternal morbidity and mortality. This analysis is part of a larger, prospective study in Nawanyago and other locations in Uganda.

### Conclusion

Increasing the number of women in the developing world who give birth with the assistance of a skilled attendant remains an important global public health priority. Our study demonstrates promising initial data that the introduction of a low cost, antenatal ultrasound program at a low resource, rural health care clinic in Sub-Saharan Africa can attract more women to come to the clinic to receive skilled antenatal care and skilled care at delivery. These well-recognized quality measures are thought to be likely to directly impact maternal and neonatal mortality and morbidity. Appropriate utilization of technology to build demand for health care resources is a promising avenue for improving maternal health outcomes in the developing world and worthy of further investigation as the global public health community continues to meet the challenges of the Millennium Development Goals.
